# Supplementary figures and images for: Population risk factors for severe disease and mortality in COVID-19: A global systematic review and meta-analysis
Source: PLoS One. 2021 Mar 4;16(3):e0247461. doi: 10.1371/journal.pone.0247461 (PMC7932512; doi:10.1371/journal.pone.0247461)

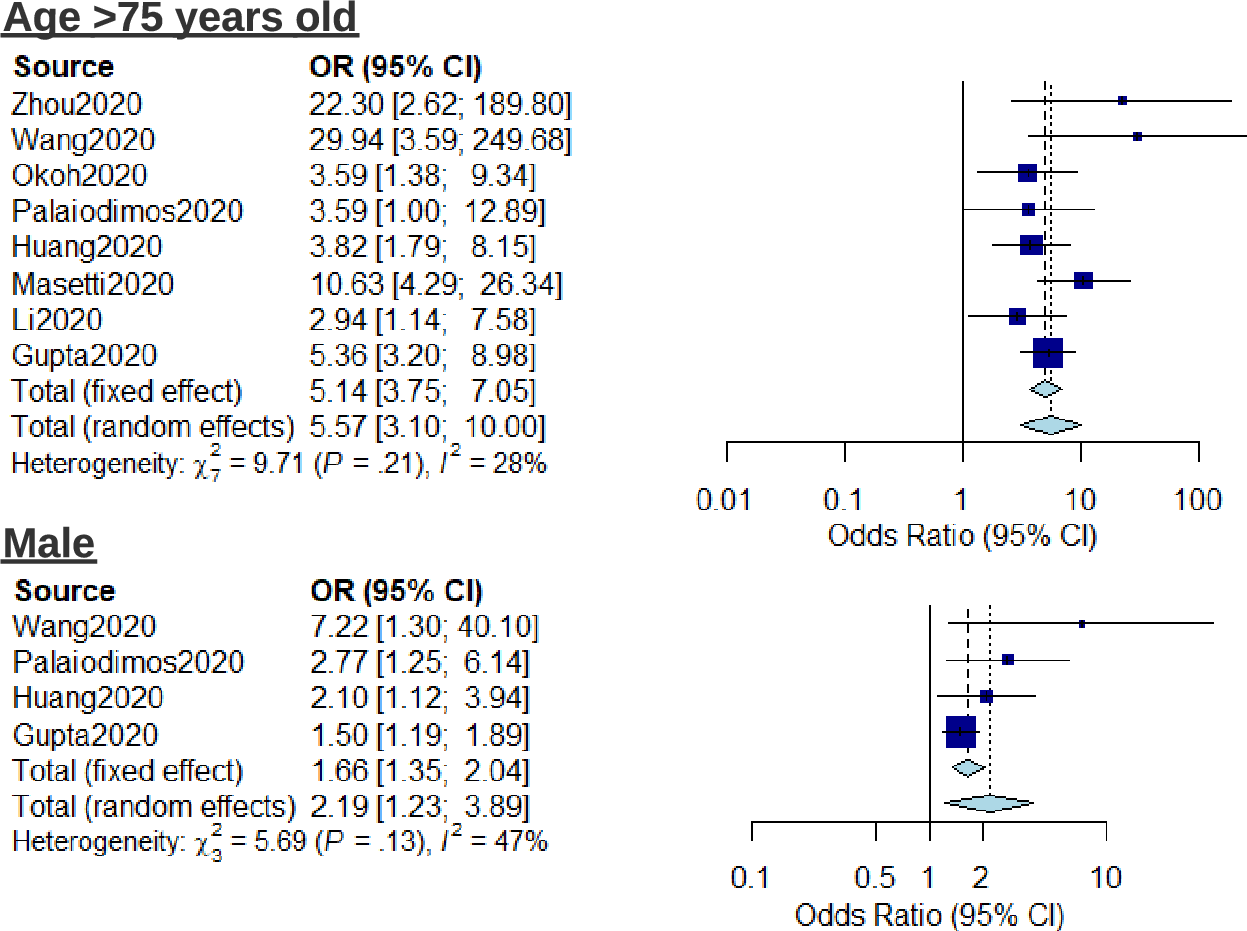

Supplement: S1 Fig — (TIF) [file pone.0247461.s002.tif]

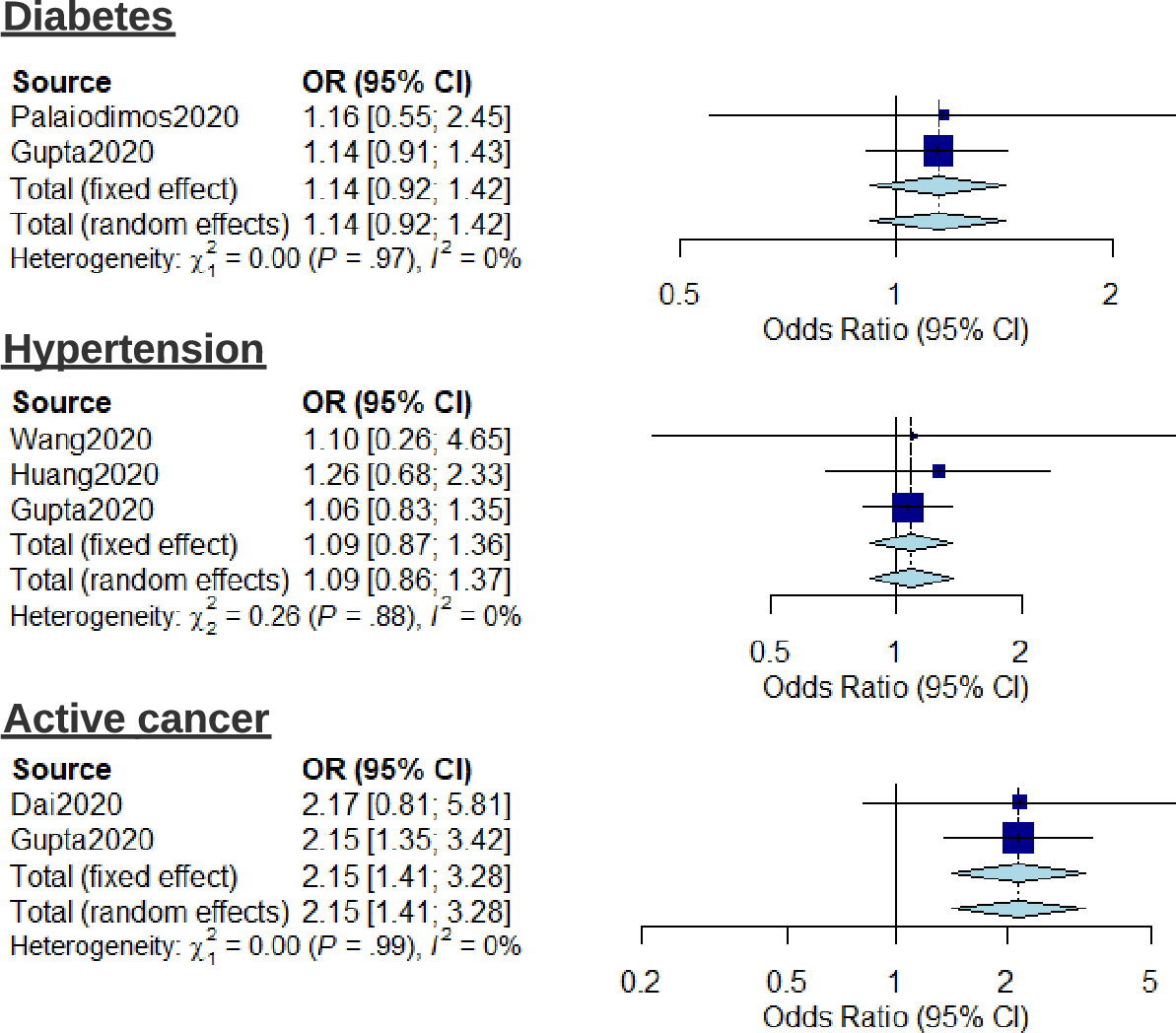

Supplement: S2 Fig — (TIF) [file pone.0247461.s003.tif]

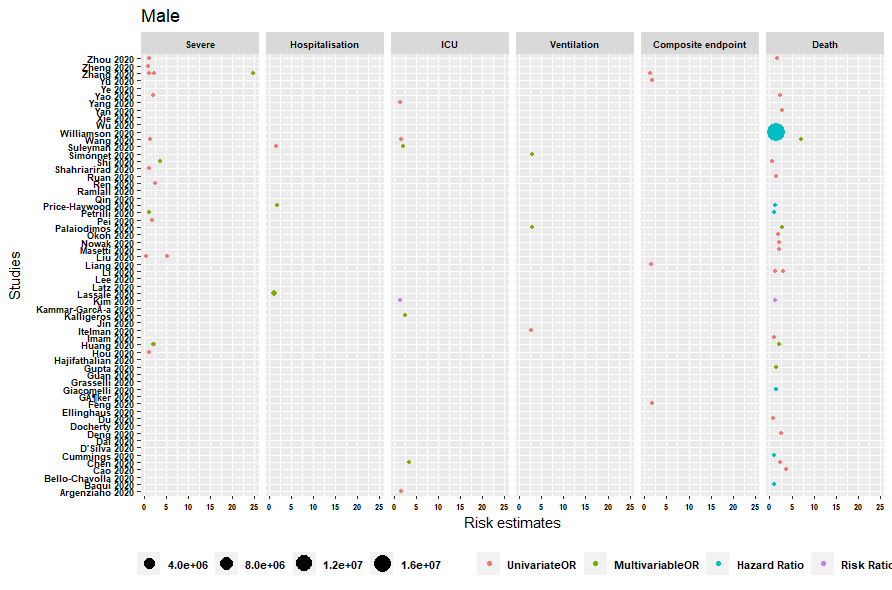

Supplement: S3 Fig — Size of the circle indicates sample size represented. (TIF) [file pone.0247461.s004.tif]

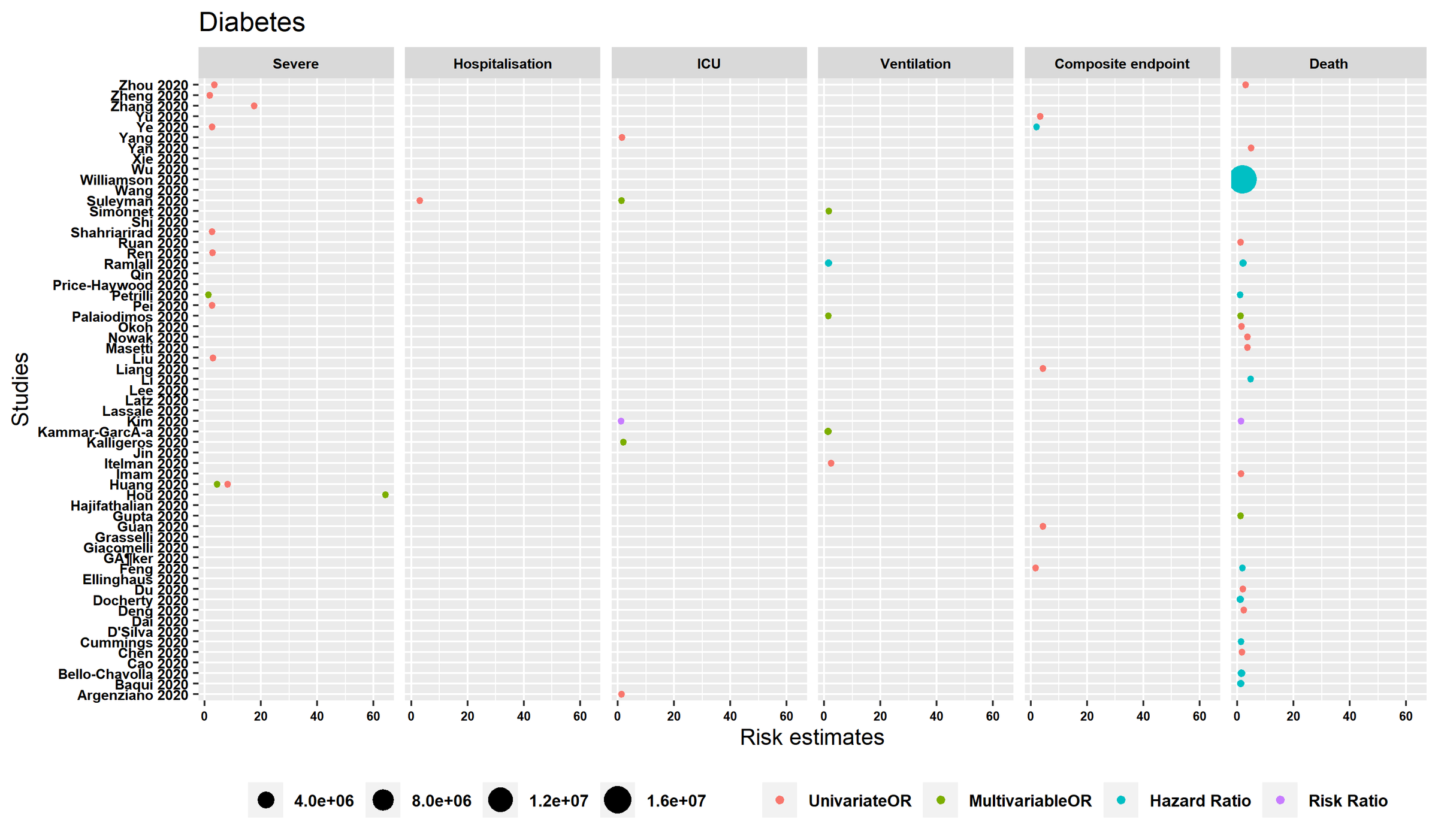

Supplement: S4 Fig — Size of the circle indicates sample size represented. (TIF) [file pone.0247461.s005.tif]

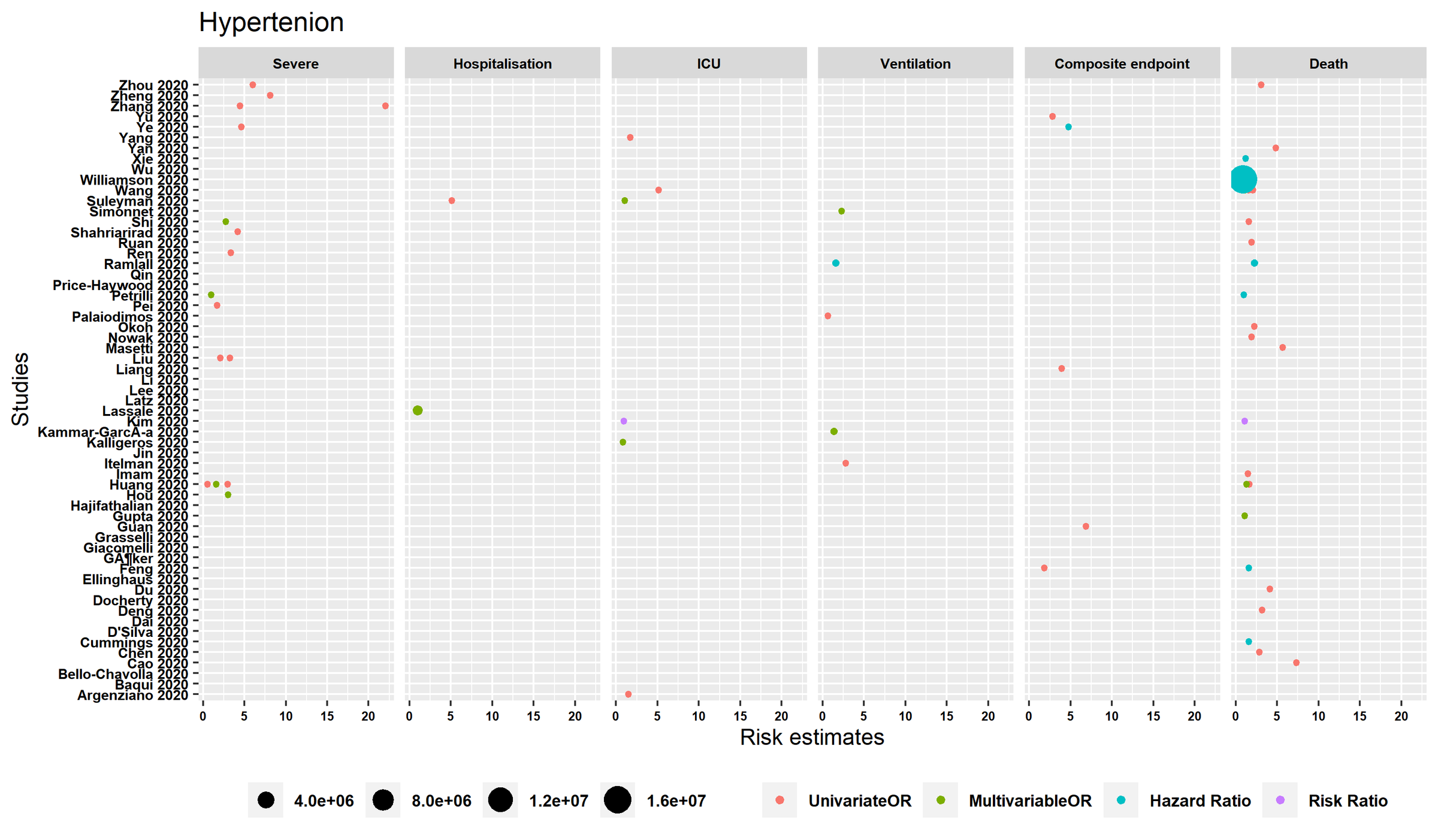

Supplement: S5 Fig — Size of the circle indicates sample size represented. (TIF) [file pone.0247461.s006.tif]
