# Supplementary material for: Population risk factors for severe disease and mortality in COVID-19: A global systematic review and meta-analysis
Source: PLoS One. 2021 Mar 4;16(3):e0247461. doi: 10.1371/journal.pone.0247461 (PMC7932512; doi:10.1371/journal.pone.0247461)
Supplement: S1 Table — (DOCX) [file pone.0247461.s007.docx]

| **S1 Table.** Systematic literature review search terms and strategy. |
| --- |
| **Inclusion Criteria** |
| - Original research article; - COVID-19 confirmed patients; - Outcome is serious disease OR survival OR one of the manifestations of severe disease (MODS, ARDS, Cardiomyopathy, Need for ventilator OR ICU care); - Includes risk estimates of association with baseline characteristics OR clinical symptoms (studies with simple proportions among disease can use the original frequencies to calculate Odds ratios); - Published since Jan 1st 2020; ; - Data reported include individual characteristics, symptoms, comorbidities and/or basic clinical measurements |
| **Exclusion Criteria** |
| - Narrative/scoping literature reviews; - Case-reports and case series including less than 100 COVID-19 patients; - Abstract only/case reports/Editorials/author responses; - Data with only lab/imaging-based parameters or data points unavailable to a patient at home; - Paediatric population (<16 years old); - Repeated data; - Clinical trials; - Pregnant women or any other exclusive homogeneous population |
| **Search terms for PubMed (18/06/2020)** |
| ((((COVID-19[Supplementary Concept]) AND ((ventilator[Title/Abstract] OR ICU[Title/Abstract] OR intensive care[Title/Abstract] OR mortality[Title/Abstract] OR prognosis[Title/Abstract] “MODS”[Title/Abstract] OR ARDS[Title/Abstract] OR severity[Title/Abstract] OR prognosis[Title/Abstract] OR hospitalis*[Title/Abstract] OR hospitaliz*[Title/Abstract] OR “respiratory failure”[Title/Abstract] OR intubation[Title/Abstract] OR ventilation[Title/Abstract] OR admission*[Title/Abstract] OR admitted[Title/Abstract] OR "critical care"[Title/Abstract] OR "critical cases"[Title/Abstract] OR severe)[Title/Abstract])) AND ((clinical[Title/Abstract] OR symptom*[Title/Abstract] OR characteristic*[Title/Abstract] OR comorbidit*[Title/Abstract] OR “co morbidit*”[Title/Abstract] OR risk[Title/Abstract] OR predict*)[Title/Abstract])) NOT ((pediatric*[Title/Abstract] OR paediatric*[Title/Abstract] OR child*)[Title/Abstract])) AND (("2020/01/01"[Date - Publication] : "3000"[Date - Publication])) Filters: English Sort by: Most Recent |
| **Search terms for Scopus (09/07/2020)** |
| (TITLE-ABS-KEY ( ncov* OR coronavirus OR "SARS-CoV-2" OR covid-19 OR covid ) AND TITLE-ABS-KEY ( ventilator OR icu OR intensive AND care OR mortality OR prognosis "MODS" OR ards OR severity OR prognosis OR hospitalis* OR hospitaliz* OR "respiratory failure" OR intubation OR ventilation OR admission* OR admitted OR "critical care" OR "critical cases" ) AND TITLE-ABS-KEY ( clinical OR symptom* OR characteristic* OR comorbidit* OR "co morbidit*OR risk OR predict* ) AND NOT TITLE-ABS-KEY ( pediatric* OR paediatric* OR child* ) ) AND DOCTYPE ( ar OR re ) AND PUBYEAR > 2019 |
