# Supplementary material for: Population risk factors for severe disease and mortality in COVID-19: A global systematic review and meta-analysis
Source: PLoS One. 2021 Mar 4;16(3):e0247461. doi: 10.1371/journal.pone.0247461 (PMC7932512; doi:10.1371/journal.pone.0247461)
Supplement: S1 File — (DOCX) [file pone.0247461.s008.docx]

Protocol: Risk factors for severe disease and mortality in COVID-19: A global systematic review and meta-analysis

Adam Booth* ^1^ & Angus Bruno Reed* ^1^, Sonia Ponzo ^1^, Arrash Yassaee ^1^, Mert Aral ^1^, David Plans ^1^, Alain Labrique ^2^, Diwakar Mohan ^2^

* These authors contributed equally.

1. Huma Therapeutics Limited, London, England.
2. Johns Hopkins Bloomberg School Public Health, Baltimore, MD, United States.

**Rationale**

COVID-19 clinical presentation is heterogeneous, ranging from asymptomatic to severe cases. The percentage of patients requiring hospitalisation is unclear, due to a lack of comprehensive testing and mild symptoms in a large proportion of patients. Among hospitalised patients, 10-20% of patients are admitted to intensive care (ICU); 3-10% require intubation and use of a mechanical ventilator; 2-5% die (Guan et al​, 2020). The main risk factors seem to be older age, male sex and pre-existing health conditions including chronic pulmonary disease, cardiovascular disease/hypertension and diabetes (Zhang et al. 2020). By collecting variables associated with risk of severe outcome, it may be possible to develop a risk model that predicts the likelihood to severe outcome. Understanding this information will support individuals and healthcare providers in managing the large number of cases and assigning resources accordingly. Many of these variables can be collected by individuals and/or clinicians in a remote setting. Collecting this information remotely would further reduce the risk of spreading COVID-19 across vulnerable patient populations and empower individuals to make informed decisions based on their personal risk profile.

While there are a number of early publications relating to risk factors for COVID-19 infection, initial data was driven primarily by data derived from hospitals in China. Given the global spread of COVID-19 it is important to consider if the risk profile for developing severe disease is shared across multiple geographies.

As such, the aim of our study is to fill this gap by systematically mapping all the available evidence on the association of various clinical variables with risk for multiple clinical outcomes in patients with COVID-19. We also examine for potential biases and we extensively search for potential overlapping studies that distorts the existing evidence.

##

## **Anticipated or actual start date**

10 June 2020

## **Anticipated completion date**

20 Nov 2020

## **Funding sources/sponsors**

## This research was funded by Huma Therapeutics Limited.

##

## **Conflicts of interest.**

A.B, A.B.R., S.P., D.P., M.A, are employees of Huma Therapeutics Ltd. D.M & AL declare that they have no conflict of interests to report.

## **Review question**

The aim of this study is to construct a risk score to indicate adverse clinical outcomes in patients with COVID-19 based on individual characteristics, comorbidities​, symptoms and basic clinical observations​.

**Search Strategy**

Relevant studies published between January 1, 2020 up to 16th June 2020, and in English language, will be included. Databases searched PubMed and medRxiv.

Additionally we will conduct a thorough hand search of the literature and review references of included papers in our systematic review to minimize likelihood our search terms did not identify all relevant papers.

Excluding factors:

- Narrative reviews
- Case-reports and case series including less than 100 COVID-19 patients.
- Multiple manuscripts of the same data
- Abstract only/case reports/Editorials/author responses
- Data with only lab/imaging-based parameters or data points unavailable to a patient at home
- Pediatric population (<16 years old)
- Clinical trials
- Homogeneous population with exclusion criteria

**P** – Patient, Problem or Population **- COVID-19**

**I** – Intervention **- NONE**

**C** – Comparison **- risk factor/symptom/variable presence**

**O** – Outcome(s) **- severe vs not severe**

**PubMed**

((((COVID-19[Supplementary Concept]) **AND** ((ventilator[Title/Abstract] OR ICU[Title/Abstract] OR intensive care[Title/Abstract] OR mortality[Title/Abstract] OR prognosis[Title/Abstract] “MODS”[Title/Abstract] OR ARDS[Title/Abstract] OR severity[Title/Abstract] OR prognosis[Title/Abstract] OR hospitalis*[Title/Abstract] OR hospitaliz*[Title/Abstract] OR “respiratory failure”[Title/Abstract] OR intubation[Title/Abstract] OR ventilation[Title/Abstract] OR admission*[Title/Abstract] OR admitted[Title/Abstract] OR "critical care"[Title/Abstract] OR "critical cases"[Title/Abstract] OR severe)[Title/Abstract])) **AND** ((clinical[Title/Abstract] OR symptom*[Title/Abstract] OR characteristic*[Title/Abstract] OR comorbidit*[Title/Abstract] OR “co morbidit*”[Title/Abstract] OR risk[Title/Abstract] OR predict*)[Title/Abstract])) **NOT** ((pediatric*[Title/Abstract] OR paediatric*[Title/Abstract] OR child*)[Title/Abstract])) **AND** (("2020/01/01"[Date - Publication] : "3000"[Date - Publication]))

**Scopus**

(TITLE-ABS-KEY ( ncov* OR coronavirus OR "SARS-CoV-2" OR covid-19 OR covid ) AND TITLE-ABS-KEY ( ventilator OR icu OR intensive AND care OR mortality OR prognosis "MODS" OR ards OR severity OR prognosis OR hospitalis* OR hospitaliz* OR "respiratory failure" OR intubation OR ventilation OR admission* OR admitted OR "critical care" OR "critical cases" ) AND TITLE-ABS-KEY ( clinical OR symptom* OR characteristic* OR comorbidit* OR "co morbidit*OR risk OR predict* ) AND NOT TITLE-ABS-KEY ( pediatric* OR paediatric* OR child* ) ) AND DOCTYPE ( ar OR re ) AND PUBYEAR > 2019

**WebOfScience**

TI = (( ncov* OR coronavirus OR "SARS-CoV-2" OR covid-19 OR covid) AND ( ventilator OR icu OR "intensive care" OR mortality OR prognosis OR "MODS" OR ards OR severity OR prognosis OR hospitalis* OR hospitaliz* OR "respiratory failure" OR intubation OR ventilation OR admission* OR admitted OR "critical care" OR "critical cases") AND ( clinical OR symptom* OR characteristic* OR comorbidit* OR "co morbidit*" OR risk OR predict* ) ) OR AB = (( ncov* OR coronavirus OR "SARS-CoV-2" OR covid-19 OR covid) AND ( ventilator OR icu OR "intensive care" OR mortality OR prognosis OR "MODS" OR ards OR severity OR prognosis OR hospitalis* OR hospitaliz* OR "respiratory failure" OR intubation OR ventilation OR admission* OR admitted OR "critical care" OR "critical cases") AND ( clinical OR symptom* OR characteristic* OR comorbidit* OR "co morbidit*" OR risk OR predict* ) ) OR AK = (( ncov* OR coronavirus OR "SARS-CoV-2" OR covid-19 OR covid) AND ( ventilator OR icu OR "intensive care" OR mortality OR prognosis OR "MODS" OR ards OR severity OR prognosis OR hospitalis* OR hospitaliz* OR "respiratory failure" OR intubation OR ventilation OR admission* OR admitted OR "critical care" OR "critical cases") AND ( clinical OR symptom* OR characteristic* OR comorbidit* OR "co morbidit*" OR risk OR predict* ) ) NOT (TI = ( pediatric* OR paediatric* OR child* ) OR AB = ( pediatric* OR paediatric* OR child* ) OR AK = ( pediatric* OR paediatric* OR child* ) OR KP = ( pediatric* OR paediatric* OR child* ))

Refined by: DOCUMENT TYPES: ( ARTICLE OR EARLY ACCESS OR REVIEW ) AND PUBLICATION YEARS: ( 2020 )

Timespan: Year to date. Indexes: SCI-EXPANDED, SSCI, A&HCI, CPCI-S, CPCI-SSH, BKCI-S, BKCI-SSH, ESCI, CCR-EXPANDED, IC.

## Intervention(s), exposure(s)

We will consider all reported cases of patients with COVID-19 and their clinical outcomes of severe (additional respiratory support, admission to ICU, and/or death) vs. mild COVID-19 infection.

## Comparator(s)/control

Patients with and without presence of risk factors identified below:

**Individual characteristics:​** Age, sex, ethnicity, BMI, smoking status, blood type

**Comorbidities​:** Cardiovascular disease ; Heartfailure ; Stroke ; Diabetes ; Hypertension ; Chronic kidney disease ; Chronic lung disease ; Chronic liver disease ; Active cancer ; Other

**Symptoms at onset/admission**: ​cough, fever, headache, fatigue, sputum production, myalgia (muscle pains), dyspnea, nausea, diarrhoea, gastro-intestinal, hemoptysis, loss of smell/taste

**Clinical observations​ at onset/admission:** temperature, blood pressure, respiratory rate, heart rate, oxygen saturation.

## Types of study to be included

We will include observational studies (including cross sectional, cohort and case–series studies) for the assessment of outcomes.

## Context

Studies in a hospital or outpatient setting

## Main outcome

The outcomes of patients with presence of demographics/anthropometric, comorbidities, symptoms or basic clinical variables and those without will be compared and a meta-analysis (where possible) will be done to report strength of association (ORs).

## Additional outcome

Development of a risk profile score in which presence or absence of multiple variables identified through main outcome, indicate risk of severe outcome for an individual.

Assess differences in geographic risk profile of severe COVID-19 across asia, europe and america.

## Data extraction (selection and coding).

Working in pairs, two authors will independently review titles and abstracts to include/exclude articles based on defined criteria. Any doubt over inclusion/exclusion will be discussed in a consensus decision, led by the PI.

Full-text review will be extracted from papers that have been included during screening, Working in pairs, two authors will independently review titles and abstracts to include/exclude articles based on defined criteria. Any doubt over inclusion/exclusion will be discussed in a consensus decision, led by the PI. Studies with missing, unclear, or incomplete data will be excluded from the review.

The following information will be extracted from each selected article: author, publication year, article title, location of study, COVID-19 case identification, study type (e.g. primary research, review, etc), peer review status, quality assessment & total sample size. Extracted data will include sample demographics (age, sex, ethnicity), obesity/BMI status, smoking status, blood type, any existing co-morbidities, symptoms, basic clinical variables (e.g. heart rate, respiration rate and oxygen saturation) and their clinical outcomes of severe (additional respiratory support, admission to ICU, and death) vs. mild COVID-19 infection.

Software specifically developed for systematic review will be used in all phases of the review (Covidence).

## Risk of bias (quality) assessment

Critical appraisal of articles will be performed by the reviewer during full-text screening using the Newcastle-Ottawa Scale to assess the methodological quality of each article.

## Strategy for data synthesis.

The outcomes of patients with presence of demographics/anthropometric, comorbidities, symptoms or basic clinical variables and those without will be compared and a meta-analysis (where possible) will be done to report strength of association (ORs).

## Dissemination.

A manuscript will be submitted to a leading journal in this field.
