# Supplementary material for: Population risk factors for severe disease and mortality in COVID-19: A global systematic review and meta-analysis
Source: PLoS One. 2021 Mar 4;16(3):e0247461. doi: 10.1371/journal.pone.0247461 (PMC7932512; doi:10.1371/journal.pone.0247461)
Supplement: Attachment — Submitted filename: Response to reviewers.docx [file pone.0247461.s010.docx]

Response to the Editor and the Reviewers:

Dear Prof. Giordano Madeddu,

We are grateful to the reviewers and yourself for your hard work on our behalf. We have now addressed all the reviewers’ comments below and in the manuscript (changes are highlighted in yellow in a version of the manuscript uploaded as Supplementary Materials). We are confident these suggested revisions have substantially improved the manuscript and hope you agree.

On behalf of all authors,

David Plans

General points:

1. Please ensure that your manuscript meets PLOS ONE's style requirements, including those for file naming. The PLOS ONE style templates can be found at

<https://journals.plos.org/plosone/s/file?id=wjVg/PLOSOne_formatting_sample_main_body.pdf>

<https://journals.plos.org/plosone/s/file?id=ba62/PLOSOne_formatting_sample_title_authors_affiliations.pdf>

2. Thank you for stating the following financial disclosure:

"This research was funded by Huma Therapeutics Ltd. "

Please state what role the funders took in the study. If the funders had no role, please state: "The funders had no role in study design, data collection and analysis, decision to publish, or preparation of the manuscript."

If this statement is not correct you must amend it as needed.

Please include this amended Role of Funder statement in your cover letter; we will change the online submission form on your behalf.

3. Thank you for stating the following in the Competing Interests section:

"A.B, A.B.R., S.P., D.P., A.Y., M.A., are employees of Huma Therapeutics Ltd. D.M & AL declare that they have no conflict of interests to report. "

Please confirm that this does not alter your adherence to all PLOS ONE policies on sharing data and materials, by including the following statement: "This does not alter our adherence to PLOS ONE policies on sharing data and materials.” (as detailed online in our guide for authors http://journals.plos.org/plosone/s/competing-interests). If there are restrictions on sharing of data and/or materials, please state these. Please note that we cannot proceed with consideration of your article until this information has been declared.

Please include your updated Competing Interests statement in your cover letter; we will change the online submission form on your behalf.

Please know it is PLOS ONE policy for corresponding authors to declare, on behalf of all authors, all potential competing interests for the purposes of transparency. PLOS defines a competing interest as anything that interferes with, or could reasonably be perceived as interfering with, the full and objective presentation, peer review, editorial decision-making, or publication of research or non-research articles submitted to one of the journals. Competing interests can be financial or non-financial, professional, or personal. Competing interests can arise in relationship to an organization or another person. Please follow this link to our website for more details on competing interests: http://journals.plos.org/plosone/s/competing-interests

Reviewer 1:

Reviewer #1: This is an interesting paper that aims at synthesizing the published evidence on relation between patients' characteristics and COVID-19 outcomes, since the beginning of pandemic until July 2020.

The focus of my review is on the methods that need some clarifications.

1. There are inconsistencies between the main text and the Supplemental material. Search strategy include PubMed, EMBASE and Web of Science in the main text, and PubMed and Scopus in Supplemental Table 1.

In Supplemental Table 1, PICO contains a description of the search terms, whereas it should report criteria for inclusion and exclusion, type of study design and choice of extracted data. The search texts for EMBASE and Web of Science are missing.

Finally, if I use [Supplemental Concept] for COVID-19 in PubMed, I just retrieve 18 articles, without using any limitation. You may have intended “MeSH”?

Using this description, I should be able to retrieve the same 2122 articles the authors found in their search, so some issue has occurred in this part of the report.

**Authors**: We thank the reviewer for their feedback on our manuscript and bringing this error on searched databases to the authors’ attention. This error was propagated from the initial protocol in which EMBASE and Web of Science were considered. The search strategy used in the manuscript is as described in the supplementary material, PubMed and SCOPUS were used to identify relevant literature. Web Of Science was searched, however, due to high volume of non-relevant literature the decision was made to remove this database from the study.

Inclusion and exclusion criteria is now listed in supplementary Table 1.

The search strategy COVID-19 [Supplementary Concept] was, at the time of search, a MeSH Concept ([NIH](https://id.nlm.nih.gov/mesh/C000657245.html)). When searching this field again (20/01/2021), we note the same outcome as the reviewer highlights. It appears the COVID-19 [Supplementary Concept] is now an obsolete term or has been replaced. COVID-19 has been a rapidly evolving topic, with changing guidance and understanding also reflected in academia. The search terms reported in the supplementary material are true to the search carried out at the time of search in PubMed (18/06/2020). To provide further evidence for this, we enclose the preliminary search strategy for identification of key variables in the search terms (15/06/2020).

| **Search** | **Search identification of key variables** | **Results**  **15/06/20** |
| --- | --- | --- |
| 1 AND 2 AND 3 AND 5 NOT 4 | ((((COVID-19[Supplementary Concept]) AND ((ventilator[Title/Abstract] OR ICU[Title/Abstract] OR intensive care[Title/Abstract] OR MODSOR ARDS[Title/Abstract] OR severity[Title/Abstract] OR prognosis[Title/Abstract] OR hospitalis*[Title/Abstract] OR hospitaliz*[Title/Abstract] OR intubation[Title/Abstract] OR ventilation[Title/Abstract] OR admission*[Title/Abstract] OR admitted[Title/Abstract] OR "critical care"[Title/Abstract] OR "critical cases"OR severe)[Title/Abstract])) AND ((clinical[Title/Abstract] OR symptom*[Title/Abstract] OR characteristic*[Title/Abstract] OR comorbidit*[Title/Abstract] OR “co morbidit*”[Title/Abstract] OR "risk factors"[Title/Abstract] OR predict*)[Title/Abstract])) NOT ((pediatric*[Title/Abstract] OR paediatric*[Title/Abstract] OR child*)[Title/Abstract])) AND (("2020/01/01"[Date - Publication] : "3000"[Date - Publication])) | 1,401 |
| 2 AND 3 AND 5 NOT 4 | (((ventilator[Title/Abstract] OR ICU[Title/Abstract] OR intensive care[Title/Abstract] OR MODSOR ARDS[Title/Abstract] OR severity[Title/Abstract] OR prognosis[Title/Abstract] OR hospitalis*[Title/Abstract] OR hospitaliz*[Title/Abstract] OR intubation[Title/Abstract] OR ventilation[Title/Abstract] OR admission*[Title/Abstract] OR admitted[Title/Abstract] OR "critical care"[Title/Abstract] OR "critical cases"OR severe[Title/Abstract]) AND ((clinical[Title/Abstract] OR symptom*[Title/Abstract] OR characteristic*[Title/Abstract] OR comorbidit*[Title/Abstract] OR “co morbidit*”[Title/Abstract] OR "risk factors"[Title/Abstract] OR predict*[Title/Abstract])) NOT (pediatric*[Title/Abstract] OR paediatric*[Title/Abstract] OR child*[Title/Abstract])) AND (("2020/01/01"[Date - Publication] : "3000"[Date - Publication])) | 47,203 |
| 1 AND 3 AND 5 NOT 4 | ((COVID-19[Supplementary Concept]) AND ((clinical[Title/Abstract] OR symptom*[Title/Abstract] OR characteristic*[Title/Abstract] OR comorbidit*[Title/Abstract] OR “co morbidit*”[Title/Abstract] OR "risk factors"[Title/Abstract] OR predict*)[Title/Abstract])) NOT ((pediatric*[Title/Abstract] OR paediatric*[Title/Abstract] OR child*[Title/Abstract])) AND (("2020/01/01"[Date - Publication] : "3000"[Date - Publication])) | 1,589 |
| 1 AND 2 AND 3 AND 5 | (((COVID-19[Supplementary Concept]) AND ((ventilator[Title/Abstract] OR ICU[Title/Abstract] OR intensive care[Title/Abstract] OR MODSOR ARDS[Title/Abstract] OR severity[Title/Abstract] OR prognosis[Title/Abstract] OR hospitalis*[Title/Abstract] OR hospitaliz*[Title/Abstract] OR intubation[Title/Abstract] OR ventilation[Title/Abstract] OR admission*[Title/Abstract] OR admitted[Title/Abstract] OR "critical care"[Title/Abstract] OR "critical cases"OR severe)[Title/Abstract])) AND ((clinical[Title/Abstract] OR symptom*[Title/Abstract] OR characteristic*[Title/Abstract] OR comorbidit*[Title/Abstract] OR “co morbidit*”[Title/Abstract] OR "risk factors"[Title/Abstract] OR predict*)[Title/Abstract])) AND (("2020/01/01"[Date - Publication] : "3000"[Date - Publication])) | 1,610 |

2. Publication bias. Please comment on publication bias: the funnel plot seems to indicate the presence of publication bias, at least visually. If a formal analysis has been performed, please report.

**Authors**: While funnel plot asymmetry was not assessed by the Egger’s test, analysis of publication bias was performed in the form of a p-curve analysis (see below). From this investigation, we deemed that p-hacking was not occurring and therefore did not report further.

As the reviewer comments, there is asymmetry visualised in the funnel plot. This reporting has been expanded in text of the Publication Bias section:

*“The funnel plot showed a somewhat asymmetrical distribution, which may be explained by the small number studies, therefore high probability that deviations in funnel shape occur due to chance. Given the presence of high heterogeneity (Table 1) and spread of study quality scores, one can conclude that study heterogeneity may be a significant factor.”*

3. Through the text, odds ratios (OR) are followed by two numbers in brackets. Please state that they represent the confidence interval (95% CI I presume). In Table 2, crude and adjusted estimates should be accompanied by their CIs, so that the reader may evaluate the statistical significance of the ORs.

It would also be useful to add the number of papers reporting information included in the pooled estimates.

**Authors**: We thank the reviewers for this request for clarification. The assumption is correct, these numbers reflect 95CI. Throughout the manuscript the reporting of OR’s and corresponding 95% CI have been modified for clarity.

Table 2 has been adapted to include 95CI where applicable. The number of studies relating to the weighted mean are also reported within each cell. In-case of a single studies reporting, we report only the point estimate as no 95CI is possible.

4. In that regard, the studies included in the Forest Plots seem far less than 76. There are seemingly discrepancies between the number of studies included in Forest Plots and those with results reported in the Supplemental Tables. Please explain if a selection has been performed, and the used criteria.

**Authors**: The reviewer highlights one of the challenges in our study. Indeed, we were unable to present data in forest plots on all 76 included studies. This is due to the fact that not all studies reported the same variables, nor outcomes. We chose to report a selection of the most widely reported variables to highlight in the forest plots. The data reported in these forest plots represents available data of all studies that reported those variables. There were no criteria used for this selection.

Minor points:

-In the first sentence of Discussion, “…a large number…relate…” should be “relates”.

-please define CVD, RT-PCR and SD in the text

**Authors**: We thank the reviewer for bringing this to our attention. We have now amended the above.

Reviewer 2:

Reviewer #2: The authors conducted a review which aims to describe the relationship patient’s risk factors for severe disease and mortality in COVID-19. The research is well conducted, and it represents one of the actual hot topics in field. This is the reason why I think results should be shared with the scientific community.

There are just some minor considerations to do before publication.

1. Introduction

Please, provide some more information about the COVID-19 clinical features (f.e. major symptoms are fever, cough, dyspnea, and minor symptoms alter the smell and taste gastrointestinal symptoms, headache, and cutaneous manifestations. Please, read and use the following articles: <https://doi.org/10.1002/hed.26269> , <https://doi.org/10.1002/hed.26204> , <https://doi.org/10.26355/eurrev_202007_22291> , <https://doi.org/10.1016/S1473-3099(20)30402-3> , <https://doi.org/10.1016/S0140-6736(20)30183-5> , <https://doi.org/10.1111/jdv.16669>)

**Authors**: We thank the reviewer for their feedback on our manuscript and suggestions for papers to include in the introduction. We have expanded on the clinical features from literature with the articles highlighted in paragraph one of the introduction.

*“Common clinical features include major symptoms such as fever, cough, dyspnoea [2], and minor symptoms such as altered sense of smell and taste [3,4], gastrointestinal symptoms [5], and cutaneous manifestations [6].”*

2. Methods

Please, revise the search strategy you reported. Main text and Supplemental table 1 are different in search strategy description.

When reporting Odds Ratios, please specify you are reporting specified confidence limits between the brackets.

**Authors**: We thank the reviewer for their feedback on our manuscript and bringing this error to the authors’ attention. As described in response to review 1 above, this error was propagated from the initial protocol in which EMBASE and Web of Science were considered. The search strategy used in the manuscript is as described in the supplementary material, PubMed and SCOPUS were used to identify relevant literature. Web Of Science was searched, however, due to high volume of non-relevant literature the decision was made to remove this search strategy.

3. Results

In table 2, when reporting Odds, please also provide CI limits.

**Authors**: Table 2 has been adapted to include 95CI where applicable. The number of studies relating to the weighted mean are also reported within each cell. In-case of a single studies reporting, we report only the point estimate as no CI are possible.

4. Language and form

There are some typos thorough the text. Please, revise carefully before resubmission.

Before using abbreviations, please provide the definition when you use them for the first time.

After these minor revisions, the article will be ready to be published.

**Authors**: We have now carefully reviewed the manuscript and amended where necessary.
